# Voluntary non‐remunerated plasma collection in Denmark: Experiences from the Central Denmark Region

**DOI:** 10.1111/vox.70339

**Published:** 2026-08-03

**Authors:** Kathrine Agergård Kaspersen, Lotte Hindhede, Sabina Christensen, Cecilie Munck Bjerre, Anne Louise Pørtner, Louise Ørnskov Drechsler, Christina Mikkelsen, Erik Sørensen, Ole Birger Pedersen, Sisse Rye Ostrowski, Betina Samuelsen Sørensen, Bitten Aagaard, Mie Topholm Bruun, Bjarne Kuno Møller, Christian Erikstrup

**Affiliations:** ^1^ Department of Clinical Immunology Aarhus University Hospital Aarhus N Denmark; ^2^ Department of Clinical Immunology Copenhagen University Hospital, Rigshospitalet Copenhagen Denmark; ^3^ Novo Nordisk Foundation Center for Basic Metabolic Research, Faculty of Health and Medical Science Copenhagen University Copenhagen Denmark; ^4^ Department of Clinical Immunology Zealand University Hospital Køge Denmark; ^5^ Department of Clinical Medicine, Faculty of Health and Medical Sciences University of Copenhagen Copenhagen Denmark; ^6^ Department of Clinical Immunology Aalborg University Hospital Aalborg Denmark; ^7^ Department of Clinical Immunology Odense University Hospital Odense Denmark; ^8^ Department of Clinical Medicine Aarhus University Aarhus Denmark

**Keywords:** blood donors, plasma, plasma collection, plasma donors, plasma‐derived medicinal products, self‐sufficiency, voluntary non‐remunerated donors

## Abstract

**Background and Objectives:**

In Denmark, increasing demand for plasma‐derived medicinal products has made plasma self‐sufficiency based on voluntary, non‐remunerated donation a political priority. This report describes recent developments in plasma collection, with a focus on the Central Denmark Region.

**Materials and Methods:**

Plasma capacity was expanded progressively over time through dedicated units, increased staffing and donor‐focused initiatives in the Central Denmark Region, particularly in 2024. National and regional donation trends were described for 2016–2025. Donor characteristics and donation frequency were summarized for the Central Denmark Region. Plasma self‐sufficiency was calculated assuming an immunoglobulin yield of 5 g/kg plasma.

**Results:**

From 2016 to 2025, national plasma donations increased by 167% reaching 161,438 donations in 2025, corresponding to 65% self‐sufficiency. In the same period, plasma donations in the Central Denmark Region increased more than threefold to 41,268 corresponding to 70% self‐sufficiency.

**Conclusion:**

Plasma collection can be substantially expanded within a voluntary, non‐remunerated system. In the Central Denmark Region, dedicated infrastructure and targeted recruitment and retention were associated with a more than threefold increase in donations and 70% self‐sufficiency, achieved through a single plasma centre in a region with 1.4 million inhabitants. The opening of four additional small facilities (November 2025–April 2026) supports projected full self‐sufficiency within 1–2 years.

**Highlights**

- Plasma collection in Denmark increased substantially within a voluntary, non‐remunerated donation system, reaching 65% national self‐sufficiency in 2025.
- In the Central Denmark Region, plasma donations have exceeded whole blood donations, and the region has achieved 70% self‐sufficiency.
- Plasma supply was expanded through dedicated infrastructure and targeted donor recruitment and retention without compromising whole blood donations.

## INTRODUCTION

Plasma‐derived medicinal products (PDMPs) are essential for treating immunodeficiencies, bleeding disorders and other chronic conditions. Increasing use of immunoglobulin products has driven a continued rise in plasma demand, leading to an expansion of plasma donation activities across Europe [1]. Despite efforts to increase plasma self‐sufficiency, many European countries remain dependent on imported plasma. Approximately 37% of plasma used for PDMP production in Europe is sourced from outside Europe [2], predominantly from the United States [3]. Strengthening national and regional self‐sufficiency is therefore a shared priority of both the World Health Organization and the European Union [4, 5].

Denmark has a publicly funded, non‐profit blood donation system based on voluntary non‐remunerated donation (VNRD), meaning that donors give blood and plasma freely without financial compensation. Historically, plasma supply relied primarily on plasma recovered from whole blood donations. In response to rising demand and a political decision to achieve plasma self‐sufficiency, plasma collection has been expanded across the country.

There is broad consensus that plasma collection based on VNRD is preferable for ethical, economic and safety‐related reasons, including donor and patient safety [6]. However, despite increasing political attention, published comparative data on achieved plasma self‐sufficiency within European VNRD‐based systems remain limited. Nevertheless, reported plasma self‐sufficiency levels in 2025 were approximately 60% in Italy [7] and 46% in Belgium [8].

This brief summarizes recent experiences from Denmark, with particular focus on the Central Denmark Region. It highlights practical approaches to plasma collection, donor recruitment and donor retention that may be relevant for both policy development and planning. Importantly, the Danish experience demonstrates that increasing demand for PDMPs can be addressed within a voluntary, non‐remunerated blood donation system.

## MATERIALS AND METHODS

### Blood system and source plasma collection structure

Denmark has a population of 6,027,601 inhabitants distributed across five regions: the Capital Region, the Central Denmark Region, the North Denmark Region, the Region of Southern Denmark and the Region Zealand [9]. The healthcare system is tax‐funded, governed by the Ministry of Health, and organized at the regional level. Whole blood and source plasma collection are hospital‐based and regulated by the Danish Patient Safety Authority.

Regional blood centres are responsible for donor management, collection, testing and quality assurance of both whole blood and source plasma. Collected plasma is supplied for contract fractionation to CSL Behring [10] under a national agreement administered by Amgros I/S, the Danish public‐sector pharmaceutical procurement organization. PDMPs are subsequently procured centrally through Amgros, which also reimburses regional blood centres for plasma collection costs [11].

Denmark has maintained a national contract for plasma fractionation with CSL Behring since 2004. Following the most recent national tender, the contract was renewed in 2024 for a 4‐year period commencing in October 2025.

Donor recruitment in Denmark is uniquely managed by the Danish Blood Donor Association, a national organization primarily staffed by volunteers and functioning independently of the hospital infrastructure. The association works in close collaboration with the regional blood centres through approximately 50 local volunteer‐based donor associations distributed across the country [12]. These local units support donor recruitment and retention activities, including public outreach, awareness campaigns and communication with donors, while blood collection and donor eligibility assessments are managed by the hospital‐based blood centres.

### Donor recruitment and retention strategies in the Central Denmark Region

The Central Denmark Region serves a population of 1,383,063 inhabitants [9]. The region was the first in Denmark to establish a large, dedicated plasma centre exclusively for plasma collection for fractionation in 2020. The centre currently operates 25 plasmapheresis donation beds. Subsequently, additional four smaller plasma collection facilities were integrated into existing blood donation sites across the region, bringing the total to five plasma donation facilities by spring 2026, with 16 plasmapheresis donation beds currently operating across the four smaller sites.

As plasma collection capacity expanded in the Central Denmark Region, donor recruitment and retention activities were gradually strengthened. Workplace visits and social media‐based awareness had been used for several years, but only to a limited extent and without dedicated personnel. In 2024, dedicated staff resources were allocated specifically to plasma donor recruitment and retention, enabling these activities to be expanded and coordinated more systematically. This facilitated continuous recruitment campaigns, donor engagement initiatives and communication efforts, while also supporting the implementation of new measures such as immediate scheduling of follow‐up appointments after donation and donor milestone cards recognizing repeated donations. Expansion of plasma donation facilities within existing blood establishments supported recruitment and retention of both new and existing donors.

Recruitment of new plasma donors included:

1. Workplace visits by blood bank staff to enrol donors and collect pre‐donation blood samples.
2. Volunteer‐led recruitment at local events.
3. Awareness and sign‐up campaigns on social media.

Retention strategies for existing plasma donors included:

1. Immediate scheduling of follow‐up appointments after donation.
2. Donor milestone cards recognizing repeated donations.
3. Anniversary gifts.
4. Local celebrations at plasma donation sites (e.g., World Blood Donor Day).
5. Competitions and donor events.
6. Community‐building activities on social media platforms.
7. Text notifications informing donors when their plasma had been sent for fractionation.

These initiatives focused on reducing logistical barriers rather than introducing costly or financially driven incentives.

### Study population

The study population comprised Danish blood donors. Data on blood donations were retrieved from regional blood bank databases for the period 2016–2025 to provide an overview of temporal trends across a 10‐year period.

### Ethics statement

The study was approved by the Danish Data Protection Agency (P‐2019–99).

We used administrative register data and, according to Danish law, ethics approval is exempt for such research. The Danish Data Protection Agency, which is a dedicated ethics and legal oversight body, thus waives ethical review and approval for the use of administrative register data when no individual contact of participants is necessary and only aggregate results are included as disseminated findings. The study is therefore fully compliant with all legal and ethical requirements.

### Statistical analyses

Temporal trends in whole blood and plasma donations were described by year (2016–2025) at both the national level and in the Central Denmark Region.

For the Central Denmark Region, plasma donor characteristics were summarized by age distribution (2025) and by mean plasma donation frequency per donor over time (2016–2025), both stratified by sex.

Plasma self‐sufficiency was calculated at both the national level and for the Central Denmark Region assuming an immunoglobulin purification yield of 5 g/kg plasma. In 2024, the national demand was estimated at 231 tonnes annually [13], representing the most recent publicly available national estimate. Thus, the same demand was assumed for 2025.

The total national immunoglobulin consumption was 1154 kg in 2024 [13], corresponding to 191 g/1.000 inhabitants.

Based on regional population size, the estimated plasma demand required to achieve self‐sufficiency in the Central Denmark Region was 53 tonnes annually.

Self‐sufficiency calculations of 2025 were based on the total amount of plasma supplied for fractionation and manufacture of PDMPs relative to the estimated demand (231 tonnes).

Data on plasma supplied for fractionation and manufacture of PDMPs in 2025 were obtained from the five regional blood centres.

## RESULTS

### National plasma collection

In 2025, Denmark had approximately 180,000 registered blood donors, of whom 116,133 made at least one donation. Between 2016 and 2025, source plasma donations increased by 167%, from approximately 60,000 to 161,438 donations, whereas whole blood donations declined by 21%, from approximately 217,000 to 171,762 donations.

In 2025, 149.869 tonnes of plasma including both recovered and source plasma were delivered for fractionation, corresponding to a self‐sufficiency degree of 65%.

### Central Denmark Region

Between 2016 and 2025, plasma donations increased by more than 300%, from approximately 10,000 to 41,268 donations, while whole blood donations declined by 13%, from approximately 45,000 to 39,016 donations. Following the establishment of the dedicated plasma centre in 2020, plasma donations increased by 106%, from around 20,000 donations in 2020 to 41,268 donations in 2025.

Plasma donations exceeded whole blood donations regionally for the first time in 2025 (Figure 1) with a total of 9282 individuals donating (46% women and 54% men).

**FIGURE 1:**
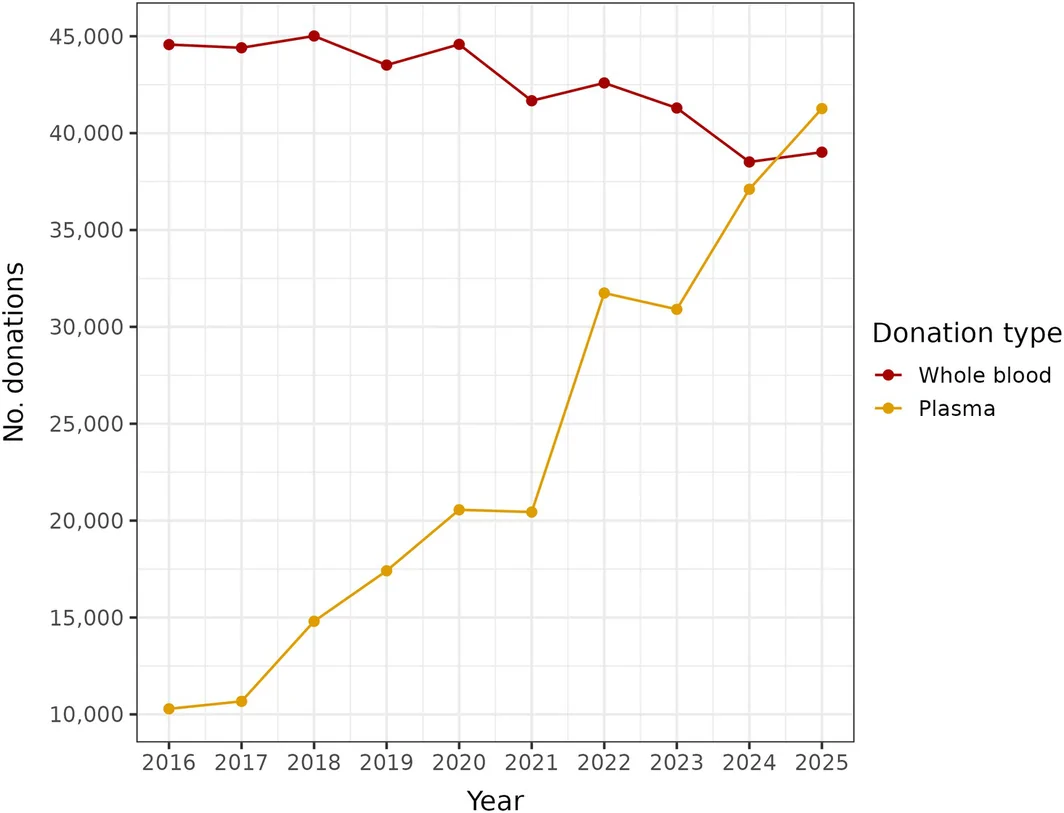
Plasma and whole blood donations in the Central Denmark Region, 2016–2025. Red: whole blood; yellow: plasma.

The median age was 41 years (interquartile range: 27–55). Plasma donors were predominantly younger than 35 years and older than 45 years (Figure 2). Mean donation frequency increased between 2016 and 2025 and was consistently higher among men than women throughout the study period (Figure 3). In 2025, the mean donation frequency reached 4.8 donations per year among men and 4.0 among women.

**FIGURE 2:**
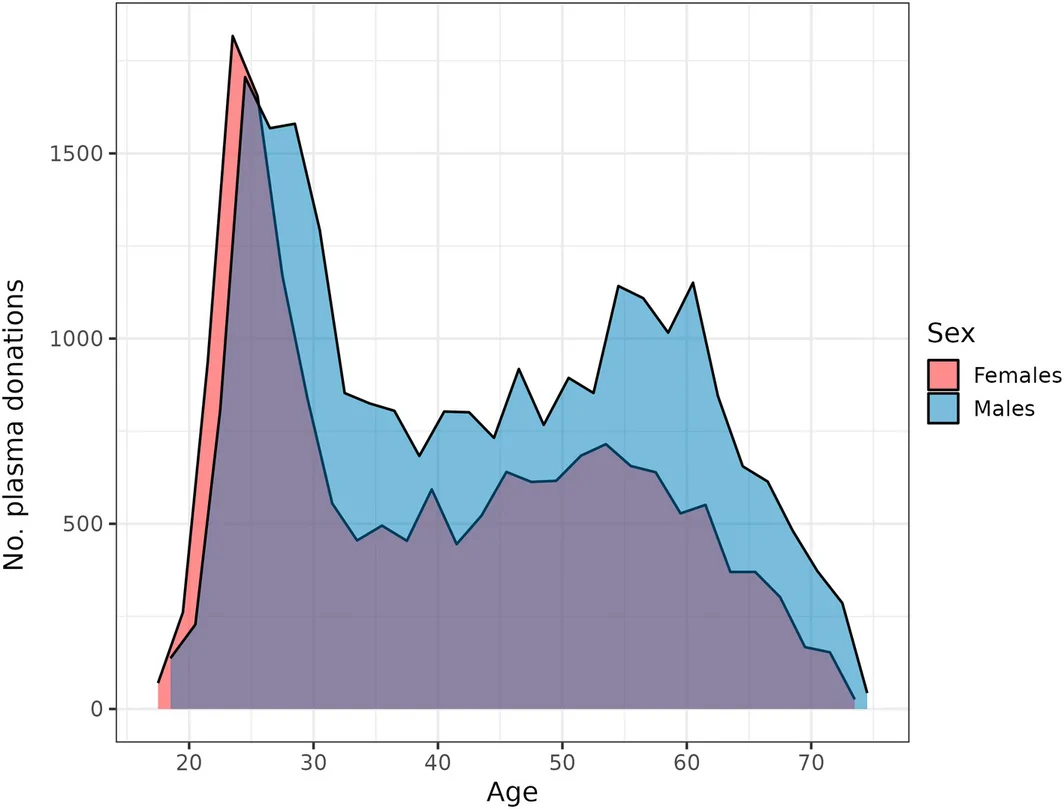
Number of plasma donations by age group and sex, 2025. Red: females; blue: males.

**FIGURE 3:**
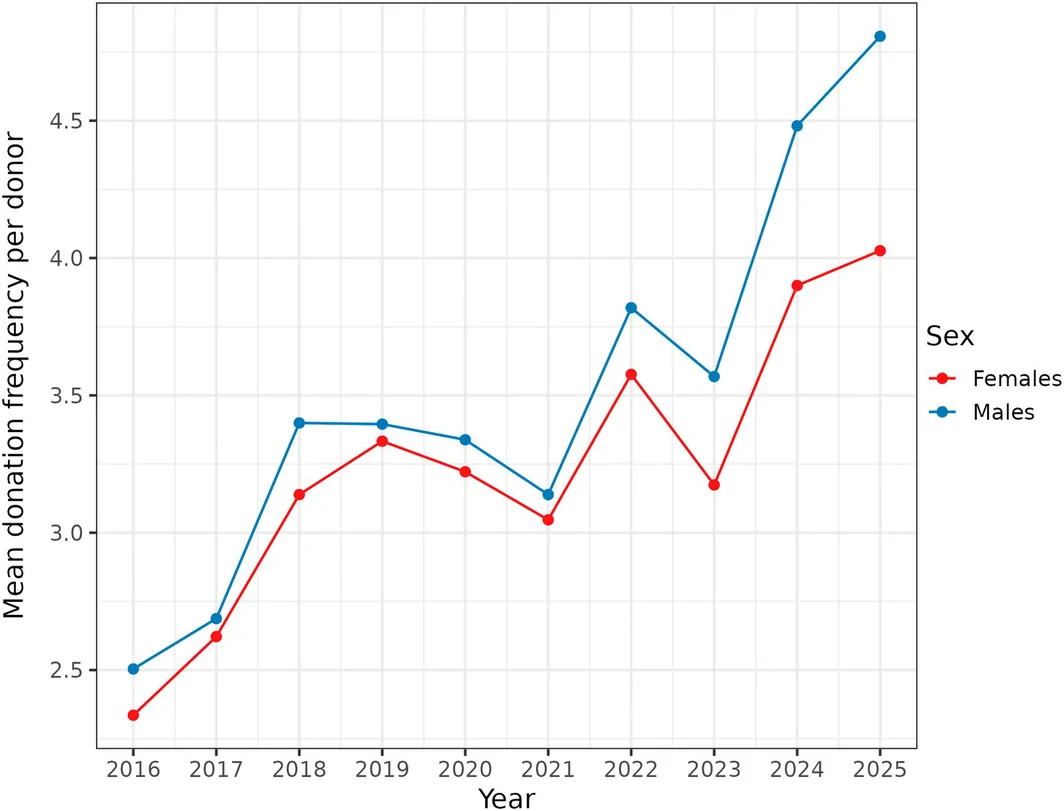
Mean plasma donation frequency per donor by sex, 2016–2025. Red: females; blue: males.

The reported amount of plasma delivered for fractionation in 2025 was 36.959 tonnes corresponding to a self‐sufficiency degree at 70%.

## DISCUSSION

Experiences from Denmark demonstrate that source plasma collection can be substantially expanded within a voluntary, non‐remunerated blood donation system. The Central Denmark Region illustrates how regionally tailored implementation can accelerate progress towards plasma self‐sufficiency through dedicated plasma centres, integration of plasma collection into existing donation sites, targeted donor recruitment and retention efforts and close collaboration between the regional blood banks and the Danish Blood Donor Association, supported by dedicated staff focusing on donor engagement and retention strategies. Of note, the observed decline in whole blood donations reflects reduced clinical demand for red blood cell components rather than limited donor availability.

Assuming a purification yield of 5 g/kg plasma, national plasma self‐sufficiency was estimated at 65%. Based on current projections, Denmark is expected to approach immunoglobulin self‐sufficiency around the time the European Union Regulation on Substances of Human Origin (SoHo) enters into force in August 2027 [13].

In the Central Denmark Region, the intensified plasma collection strategy resulted in more than 41,000 plasma donations in 2025, corresponding to a regional plasma self‐sufficiency of 70%. This was achieved prior to the planned opening of additional plasma donation facilities, expected to increase regional capacity by 40% and enable full self‐sufficiency within the next 1–2 years.

Beyond organizational strategies and infrastructure, the Danish experience may also reflect a strong societal commitment to voluntary donation. Informal donor feedback has suggested that plasma donation is often perceived as a meaningful contribution to patient care and to society more broadly [14]. Such altruistic motivation may represent an important enabling factor when scaling up plasma collection within voluntary, non‐remunerated systems. Nevertheless, evidence suggests that altruism alone does not explain donor behaviour. Accessibility of donation facilities appears to be an important factor, as increased travel distance and closure of donation centres have been associated with higher rates of donor lapse [15].

A limitation of our estimates is that they do not account for potential future increases in immunoglobulin demand, which may influence the timeline for achieving full self‐sufficiency. Furthermore, limited published data from other European countries were identified, which restricts direct comparison of plasma self‐sufficiency levels across similar voluntary donation systems.

Overall, Denmark's experience aligns with broader European policy objectives aimed at strengthening self‐sufficiency and resilience of blood and plasma supply. The upcoming EU SoHO regulation places greater emphasis on governance, coordination of plasma supply and donor safety and protection, including prevention of exploitation without discouraging donation, underscoring the relevance of practical experience from VNRD‐based systems [16].

In conclusion, plasma collection in Denmark can be substantially expanded within a voluntary, non‐remunerated blood donation system. National plasma self‐sufficiency reached 65% in 2025. The Central Denmark Region further demonstrates that dedicated plasma infrastructure and targeted donor recruitment and retention were associated with a more than threefold increase in plasma donations and achievement of 70% regional self‐sufficiency without compromising whole blood donations. These findings provide practical experience relevant to other countries seeking to strengthen domestic plasma supply.

## CONFLICT OF INTEREST STATEMENT

C.E. has received funding for other studies from Abbott Diagnostics and Novo Nordisk; no personal fees.

## Data Availability

Access to individual‐level data is governed by the Danish Authorities. Each scientific project must be approved before initiation, and approval is granted to a specific Danish research institution. Researchers at Danish research institutions may obtain the relevant approval and data. International researchers may gain data access if governed by a Danish research institution as approval and data access are required.
